# Borderline features are associated with inaccurate trait self-estimations

**DOI:** 10.1186/2051-6673-1-4

**Published:** 2014-04-10

**Authors:** Leslie C Morey

**Affiliations:** Department of Psychology, Texas A&M University, College Station, TX 77843-4235 USA

**Keywords:** Borderline personality, Self-concept, Perceptual accuracy

## Abstract

**Background:**

Many treatments for Borderline Personality Disorder (BPD) are based upon the hypothesis that gross distortion in perceptions and attributions related to self and others represent a core mechanism for the enduring difficulties displayed by such patients. However, available experimental evidence of such distortions provides equivocal results, with some studies suggesting that BPD is related to inaccuracy in such perceptions and others indicative of enhanced accuracy in some judgments. The current study uses a novel methodology to explore whether individuals with BPD features are less accurate in estimating their levels of universal personality characteristics as compared to community norms.

**Method:**

One hundred and four students received course instruction on the Five Factor Model of personality, and then were asked to estimate their levels of these five traits relative to community norms. They then completed the NEO-Five Factor Inventory and the Personality Assessment Inventory-Borderline Features scale (PAI-BOR). Accuracy of estimates was calculated by computing squared differences between self-estimated trait levels and norm-referenced standardized scores in the NEO-FFI.

**Results:**

There was a moderately strong relationship between PAI-BOR score and inaccuracy of trait level estimates. In particular, high BOR individuals dramatically overestimated their levels of Agreeableness and Conscientiousness, estimating themselves to be slightly above average on each of these characteristics but actually scoring well below average on both. The accuracy of estimates of levels of Neuroticism were unrelated to BOR scores, despite the fact that BOR scores were highly correlated with Neuroticism.

**Conclusions:**

These findings support the hypothesis that a key feature of BPD involves marked perceptual distortions of various aspects of self in relationship to others. However, the results also indicate that this is not a global perceptual deficit, as high BOR scorers accurately estimated that their emotional responsiveness was well above average. However, such individuals appear to have limited insight into their relative disadvantages in the capacity for cooperative relationships, or their limited ability to approach life in a planful and non-impulsive manner. Such results suggest important targets for treatments addressing problems in self-other representations.

## Background

Central to many theoretical accounts of borderline personality disorder (BPD) is the hypothesis that gross distortion in perceptions and attributions related to self and others represent a core mechanism for the enduring difficulties displayed by such patients. In many leading treatments for BPD, addressing such distortions is assumed to be key to therapeutic change. For example, Cognitive Therapy for BPD is based upon the assumption that such individuals have distorted and dysfunctional thoughts and beliefs about themselves and others [1], and that movement towards a “realistic appraisal of one’s circumstances will reduce the severity of accompanying distress” [2], p. 503–504. Fonagy and Bateman’s [3] Mentalization-Based Therapy (MBT) assert that individuals with BPD have developed disturbances in their internal mental representations of self and others, and MBT seeks to improve the capacity to understand one’s own perceptions and understanding of the self and others; studies of MBT have demonstrated treatment-related improvement in integration of mental representations of self and others as well as improvements in associated symptoms such as suicidality, anxiety and depression [4]. Finally, Transference Focused Psychotherapy TFP; [5, 6], is a modified psychodynamic treatment for BPD based on Kernberg’s [7] object relations model of the disorder, a model that views patients with BPD as unable to maintain a differentiated representation of self or other, and thus tending to have “limited capacity for a realistic evaluation of others” [7], p. 14 as well as having self-images which vacillate and are modally inconsistent with the way others perceive them. Although each of these different interventions are based upon different assumptions about the causal origins of distortions in self and other perception, they converge in representing the need to address these distortions as a final common pathway toward therapeutic improvement.

However, there has been limited experimental research testing the assumption that individuals with BPD demonstrate these putative distortions, and the results of these studies have been mixed. It is often found that borderline patients have strikingly negative perceptions of themselves and of others, particularly those others with attachment salience such as parents or romantic partners [8]. Nonetheless, such findings provide limited insight into the extent to which such perceptions are truly distorted or inaccurate. The most widely used experimental paradigm to study self/other distortions in BPD have involved the perception and recognition of emotion in facial expression of other people, based upon the hypothesis that impairments in emotion recognition found in disorders such as autism or schizophrenia [9, 10] might also characterize BPD. An early study by Levine et al. [11] did find that individuals with BPD were less accurate at labeling facial affects in stimulus picture sets (such as those from [12]) than healthy controls, as did a later examination by Bland et al. [13]. However, other studies reported that individuals with BPD were *more* accurate than healthy controls at detecting certain emotions [14, 15]. Follow-up investigations have attempted to clarify moderators that might explain such apparently conflicting results. Thus, poorer performance in borderline individuals has been found to be related to complexity of the facial expression [16], as well as the rapidity of the judgments involved [17]. Different findings might also be obtained when stimulus information is restricted to the eyes rather than the entire face, as Fertuck et al. [18] found greater perceptual accuracy of BPD patients relative to controls when emotion judgments were based upon the *Reading the Mind in the Eyes Test* RMET: [19]. To the extent that this literature can be summarized, it appears that BPD may be associated with subtle impairments in the recognition of basic emotion, perhaps most notable involving a negativity bias or heightened sensitivity to negative emotions [20]. However, because these experiments have been based upon stimulus sets typically developed by having actors portray certain emotional states e.g., [12], it is still unclear whether this constitutes a true test of “accuracy” as presumably these actors were not naturally experiencing the portrayed emotion at the time the stimulus pictures were taken.

There has been less experimental research on the accuracy of self-perceptions in BPD, with the most relevant findings involving the discrepancy between self- and observer-report. Klonsky et al. [21] reviewed a small number of studies that included both self- and informant-report of various personality disorder features, and concluded that the correspondence between self and informant report was generally fairly low, although assessments of BPD were not identified as being particularly discrepant relative to other personality disorder features. One such study was conducted by Zimmerman et al. [22], who reported two findings for BPD that were somewhat paradoxical in nature; although the self/informant concordance rate for BPD was higher than for any other personality disorder (kappa = .32), the prevalence estimates for BPD were dramatically different across self- and informant-interviews, with 9.1% of patient interviews resulting in a BPD diagnosis and 27.3% of informant interviews yielding a BPD diagnosis. In fact, in the Zimmerman et al. [22] study, BPD was the only personality disorder to display significant differences in prevalence across self and informant report, with much more BPD pathology reported by informants. However, that finding stands in apparent contrast to results reported by Edell et al. [23], who found that self-reports of different types of psychopathology in BPD were considerably more severe that those of a non-BPD patient group who had received comparable ratings on these same constructs by independent clinical evaluators. It is important to recognize that these involve somewhat different but interrelated questions—(a) whether self- and other-reports of BPD symptoms converge, and (b) whether self-views of patients identified as having BPD converge with other’s perceptions. Thus, although it is often found that self-ratings of BPD–relevant personality and psychopathology features diverge appreciably from those provided by informants or independent observers, the specific nature of the divergence appears to be inconsistent. Furthermore, it is not clear whether such divergences actually reflect inaccuracy or distortions in self-ratings, because informant ratings often are little more convergent with ratings from other informants than they are with patient self-ratings [21]. Furthermore, for some features, it appears that self-perceptions of BPD symptoms tend to have greater predictive validity than observer ratings of these same features [24].

The goal of this study was to explore the possibility that borderline personality features are related to distortions in perception of the self as compared to others, using a novel methodology to examine this issue. In this study, a non-clinical sample was asked to describe their personality by estimating their level on fundamental personality traits, in comparison to others (i.e., relative to the “norm”). Then, a formal assessment measure of these personality traits was administered and scored against a national, community normative sample for this measure. It was hypothesized that individuals with prominent borderline personality characteristics would be significantly less accurate in their estimate of their own personality characteristics as compared to such norms, as might be expected if such individuals demonstrated a significantly distorted self-view. In addition, the study sought to explore the impact of different specific features of BPD as well as the impact of estimating different types of traits (e.g., interpersonal vs. intrapsychic characteristics), to determine if certain aspects of BPD might be particularly associated with mis-estimation of certain types of traits.

## Method

### Participants

Participants included 104 college students enrolled in an upper-level course on personality psychology. Participation represented an optional educational research project as an opportunity to learn more about trait models of personality; 104 of the 115 students in the course (90.4%) elected to participate in the project. The average age of the sample was 21.4 years; 64.4% were women, whereas 35.6% were men. The study was reviewed and approved by the Institutional Review Board of Texas A&M University.

### Measures

#### *Personality assessment inventory borderline features (PAI BOR) scale*

The PAI [25] is a multi-scale self-report clinical inventory assessing a variety of different clinical conditions. The 24-item Borderline Features (BOR) scale was constructed with four subscales targeting different theoretical elements of the disorder as identified in previous research e.g., [26]. The four subscales include: *Affective Instability* (BOR-A), assessing poor modulation of emotional responses; *Identity Disturbance (BOR-I),* tapping uncertainty about major life issues and a general lack of purpose; *Negative Relationships (BOR-N),* reflecting a history of intense, ambivalent relationships; and *Self-Harm (BOR-S),* measuring impulsivity with an accompanying disregard for potential negative consequences. The BOR scale was normed against a census-matched national sample and uses t-scores that have a mean of 50 t and a standard deviation of 10 t in the general population. In this sample, the mean PAI-BOR score was 52.55 t (SD = 9.89), and 10 of 104 participants obtained scores of 70 t or greater (i.e., two SDs above the mean), a cutoff that has been used effectively to differentiate borderline from non–borderline participants in previous research e.g., [27]. Numerous studies have demonstrated the validity and reliability of the PAI-BOR scale among both nonclinical samples e.g., [27, 28], and clinical samples e.g., [29]. The internal consistency of the PAI-BOR scale among all participants in the sample was .85.

#### *NEO-five factor inventory (NEO-FFI)*

The NEO-FFI [30] is a 60-item questionnaire with 12 items tapping each of the higher order domains of the Five Factor Model: Neuroticism (N), Extraversion (E), Openness (O), Agreeableness (A), and Conscientiousness (C). Items require the respondent to rate the extent to which he or she concurs with a statement on a 5-point Likert-type scale ranging from “Strongly Disagree” to “Strongly Agree”. Scores on the NEO-FFI are normed against the responses from a national community sample of adults 18 and older [30]. The NEO-FFI scales correlate substantially with the more comprehensive scales from the full NEO-PI-R, tend to demonstrate good internal reliabilities [31], and account for roughly 85% of the variance in convergent validity criteria from the full instrument [30]. The internal consistencies of the NEO-FFI scales among all participants in this sample were as follows: Neuroticism (.88), Extraversion (.84), Openness (.73), Agreeableness (.81), and Conscientiousness (.86).

### Procedure

As noted previously, all participants were enrolled in an upper-level college course on personality psychology. Participation in the study was completed within a two-day period following a one-hour lecture on trait psychology, specifically on the Five-Factor Model (FFM) of personality e.g., [31]. After the lecture, students were informed that they could participate in an optional educational research project that would involve completing a personality measure of the FFM that would provide feedback about their personality profile on these five dimensions. Of the students in the class, most (90.4%) volunteered to participate.

Participation involved logging in to a password-protected course website and completing an online questionnaire. This questionnaire began with basic demographic information, and next provided a description of the five domains of the FFM using material adapted from McCrae and Costa e.g., [31]. After reading the description of each trait domain, participants were shown a picture of a normal distribution, divided into nine parts, and asked to indicate where in such a distribution they felt that they were on this trait. Thus, for example, if participants felt that they were slightly below average on Neuroticism, they were to indicate a score in a range slightly less than the mean score on the distribution for that trait. The nine ranges used were derived from stanine scores [32] which divide the normal distribution into nine standard units, with the mean being 5 and the standard deviation equivalent to 2.

After indicating their self-ratings for each of the five domains of the FFM, participants then completed the 24 items of the PAI-BOR and the 60 items of the NEO-FFI. Feedback about NEO-FFI scores were provided to participants one week after completion of their participation, and were expressed as standard normal scores (i.e., z-scores) as normed against the national community norms for the NEO-FFI [30].

### Analyses

For each participant, an “inaccuracy” score on each domain of the FFM was calculated by taking the squared difference between the NEO-FFI z-score calculated from national norms, and the estimated z-score as derived from the self-descriptive “stanine” rating provided by each participant. The five domain inaccuracy scores were then summed into a total inaccuracy score, and then these scores were correlated with the full and subscale scores from PAI-BOR.

## Results

Table 1 presents the correlations between the full and subscale scores for PAI-BOR with the participants’ scores on the NEO-FFI domains, as well as with inaccuracy scores on the individual domains as well as a total inaccuracy score. The pattern of correlation between the FFM domains and borderline features largely corresponds to what has been found in the literature e.g., [33–36]: large positive associations with N, negative associations with E, A and C, and little relationship to O.

**Table 1:** **Correlations of borderline features with FFM traits and inaccuracy of trait self-estimations**

	BOR	BORA	BORN	BORI	BORS
*NEO-FFI Scores*					
Neuroticism	.7671**	.7300**	.6223**	.7293**	.1837
Extraversion	-.5331**	-.6260**	-.4726**	-.3271**	-.1355
Openness	-.0778	.0176	-.0774	-.1780	.0001
Agreeableness	-.5716**	-.5869**	-.4155**	-.3561**	-.3604**
Conscientiousness	-.5422**	-.4970**	-.2827**	-.5120**	-.3469**
*Inaccuracy Scores*					
Neuroticism	.1591	.0401	.1820	.0896	.1973*
Extraversion	.1471	.0695	.0719	.1528	.1707
Openness	-.0077	-.0655	-.0973	.0437	.1239
Agreeableness	.3332**	.2335*	.2035*	.3686**	.2095*
Conscientiousness	.4022**	.3967**	.2745**	.2843**	.2566**
Total	.4805**	.3560**	.2879**	.4384**	.3944**

Notes: *p < .05 **p < .01.

Of greater relevance to study hypotheses are the associations with the inaccuracy scores. These associations are scaled such that positive correlations indicate individuals with higher PAI-BOR scores being less accurate in estimating their FFM trait scores, relative to others. Thus, the sizeable .48 association between BOR full scale and total inaccuracy indicates that individuals with more borderline characteristics were less accurate in estimating their levels of core personality traits than were those with fewer borderline characteristics. In particular, these individuals were inaccurate in estimating their Agreeableness and Conscientiousness scores. To further explore the nature of this mis-estimation, the estimations of the 10 study participants who scored 2 or more standard deviations above populations norms (i.e., within the clinical range) for PAI-BOR were examined. For these participants, large differences were observed between estimated and actual scores on both A and C. For Agreeableness, these participants estimated that they were slightly above average (mean estimated Z score = +0.30, SD = 2.11), but their actual NEO-FFI Agreeableness, scores placed them well below average (mean actual Z score = -1.29. SD = 1.85, paired difference t-test(9) = 3.53, p < .006). Similarly, for Conscientiousness, the high-BOR participants estimated that they were slightly above the mean (mean estimated Z score = +0.20, SD = 1.48), but their NEO-FFI Conscientiousness scores indicated again that they were well below average on this trait (mean actual Z score = -1.60, SD = 1.48, paired difference t-test(9) = 4.70, p < .001). With respect to the various different borderline features, all four PAI-BOR subscales were related to inaccuracy of estimation, with the Identity elements demonstrating the largest associations with total inaccuracy scores.

## Discussion and conclusions

The current study tested the hypothesis that borderline personality features are associated with distortions in the perception of self and other by examining the degree to which participants could estimate their level on core traits of personality, relative to the general population. This hypothesis was confirmed with an impressive relationship between BPD features and inaccuracy of estimates. This pattern of inaccuracy was typically directional on certain traits; specifically, individuals with prominent borderline characteristics greatly overestimated their levels of Agreeableness and Conscientiousness relative to others. On both traits, such individuals estimated that they were slightly above average on these characteristics, while in actuality these participants were well below average relative to community norms. These findings represents one of the first documentations of the type and degree of distortions in self-perception that have been thought to reflect a prominent feature of BPD as well as an important target for treatment.

It is also important to note that while estimates of Agreeableness and Conscientiousness tended to be considerably inaccurate among those with high PAI-BOR scores, the accuracy of estimates of Neuroticism were largely unrelated to borderline features. As found in this and many previous studies, borderline symptoms are typically highly correlated with the Neuroticism domain, and the .76 correlation obtained here makes it evident that high BOR participants also tended to score high on Neuroticism. Even so, high BOR status was unrelated to accuracy of estimates of N scores—for the most part, high BOR participants accurately perceived themselves as above average on N, while low BOR scorers estimated themselves as lower on N. Thus, the perceptual inaccuracies associated with borderline features are not merely a function of being globally unable to recognize their differences from the “norm”; although such individuals appear to understand that their emotional sensitivity is above average, they fail to appreciate their relative disadvantages in the capacity for cooperative relationships (A) or ability to approach life in a planful and non-impulsive manner (C). However, it is not clear whether this represents a failure to accurately appraise these characteristics in the self, or perhaps whether this discrepancy results from an inaccurate perception of the “norm” for such characteristics in others. Future research directed at exploring alternative elements of such distortions would be an important extension of these findings.

It is important to recognize that the study, while presenting a novel methodology for the study of self/other accuracy, has limitations. First, the reliance upon a college student sample reduces generalizability due both the age of the participants, as well as to the non-clinical status. Replicating this finding in a clinical sample of BPD patients and other appropriate control groups (both clinical and non-clinical) would be important, although it would be challenging to provide such patients with comparable material (to the instruction received by these students) on concepts like the Five Factor Model that would allow them to make informed estimates of their trait levels. Although participants scoring in the clinical range on the PAI-BOR scale demonstrated marked inaccuracy in estimating certain trait scores, these individuals comprised a small subset of this non-clinical sample. It is important to note that the sample obtained mean and standard deviation values on PAI-BOR that were quite similar to those obtained in the national, census-match normative sample for the PAI [25].

Another consideration for the current study is that the data relied exclusively on the self-report of participants. In one way, this constitutes a potential advantage of this method, because mode of information gathering is held constant, with the tested difference involving a global self-appraisal of a trait as compared to a systematic, normatively based measurement of that same trait. Thus, the study does provide a genuine “accuracy” metric in that it compares a subjective impression to an objective, data-based norm—albeit, one derived from self-report items. As such, there continues to be a need for further study of these perceptual distortions that involve comparisons of differences between borderline patients’ perceptions of self and other, as gauged against external references for those perceptions. This area is fraught with challenges, as it is difficult to establish a criterion for what is “accurate”—for example, are other, non-borderline people’s perceptions of BPD patients, which tend to be negative [37], a reasonable criterion against which to determine “accuracy”?

Despite these challenges, it is essential for the field to continue to clarify the specific nature of BPD-related cognitive/perceptual distortions of self and other, because so many of the extant treatments of this disorder identify this as a central target for change. The results of this investigation identify two promising targets for continued research—one, an interpersonal dimension (Agreeableness), and the second, a more intrapsychic dimension (Conscientiousness) that each have implications for important BPD symptoms such as interpersonal turmoil and failures to inhibit self-damaging impulses. These findings suggest that individuals with BPD may have insight into their heightened emotional responsiveness, but perhaps lack insight into their deficiencies in impulse control and empathic capacity. Continued research in this area is needed to help refine our understanding of such deficits, eventually incorporating such findings into treatments that focus upon improving the accurate appraisal of key personality characteristics of both self and of others.
